# An Emerging Model for Community Health Worker–Based Chronic Care Management for Patients With High Health Care Costs in Rural Appalachia

**DOI:** 10.5888/pcd17.190316

**Published:** 2020-02-13

**Authors:** Richard Crespo, Matthew Christiansen, Kim Tieman, Richard Wittberg

**Affiliations:** 1 Marshall University School of Medicine, Huntington, West Virginia; 2 Claude Worthington Benedum Foundation, Pittsburgh, Pennsylvania; 3 Community Health Improvement Associates, Inc, Marietta, Ohio

## Abstract

Community health workers (CHWs) can improve patients’ health by providing them with ongoing behavioral support during the health care experience, and they help decrease health care costs, especially among patients whose starting costs are high and among underserved and minority populations. We developed a CHW-based care model with the aim of improving outcomes and lowering costs for high-risk diabetes patients in rural Appalachia. Enrolled patients experienced a mean decrease in HbA_1c_ of 2.4 percentage points, and 60% or more of patients with diabetes lowered their blood glucose between baseline and 6 to 12 months after enrollment. As health care providers and patients became familiar with this model of care management, enrollment in the program accelerated.

**Summary**

**What is already known on this topic?**

There is evidence that the use of community health workers (CHWs) can improve the health of patients.

**What is added by this report?**

This model offers a unique look at the process by which insurance companies offered their resources in support of a policy using CHWs that improves public health.

**What are the implications for public health practice?**

This report demonstrates a pathway to achieve sustainability for activities performed by CHWs and that they can become a permanent part of health care services.

## Introduction

Community health workers (CHWs) are locally based, culturally competent lay health care workers who are uniquely situated to provide ongoing behavioral support to a cohort of patients in conjunction with the broader health care team (1). CHWs have shown to improve health in a wide range of chronic conditions, such as cancer (2), diabetes (3–9), cardiovascular disease (10–12), multiple medical comorbidities (13), and mental health (14). The root of their effectiveness relies on their close connection to the community, ability to influence patient behaviors, and effective interaction with the larger health care team (15,16). CHWs are powerful drivers of decreased costs, especially among patients with high starting health care costs and among underserved and minority populations (3,12,17).

With the recent emphasis on health system transformation to improve patient outcomes while decreasing costs, CHWs offer a unique opportunity to achieve these goals while also improving patient experience. The challenge in rural Appalachia, however, is no sustainable funding mechanisms exist that support CHWs. Typically, CHWs are grant-supported, and when the grant ends their employment ends.

We studied the dissemination of a community health worker–based chronic care management (CHW-Based CCM) model in rural counties in 3 central Appalachian states and the process for engaging health insurance payers to sustaining the model. This project serves rural areas of Central Appalachia in counties that the Appalachian Regional Commission designates as distressed or at risk. Appalachia has a high prevalence of chronic disease; many counties are in the fifth quintile in obesity, smoking, and related chronic diseases (18). People in Appalachia have lower access to health care and have higher rates of chronic diseases including heart disease, stroke, and diabetes. High stroke mortality rates have persisted in the southeastern United States, and a significant number of Appalachian counties are in the “stroke belt” (19).

In 2011 the Centers for Disease Control and Prevention labeled a 644-county area as the “diabetes belt,” which is geographically parallel to the stroke belt and more than one-third of which is in central and southern Appalachian counties. Using Behavioral Risk Factor Surveillance System data, we found that the residents in counties classified as distressed by the Appalachian Regional Commission were 1.4 times more likely to report diabetes than residents of nondistressed counties, regardless of race (20). A recent study found that residents of distressed and at-risk counties have a 40% to 50% lower odds of having annual foot and eye examinations and 30% lower odds of receiving diabetes education (21), which further highlights the importance of addressing barriers to health screening and preventive care.

We developed the CHW-Based CCM model in partnership with Duke University and Williamson Health and Wellness Center, under a Centers for Medicare and Medicaid Services Health Care Innovation Award in 2012, with the aim of improving outcomes and lowering costs for high-risk diabetes patients. Williamson Health and Wellness Center is in rural Mingo County, West Virginia. The model demonstrated a mean hemoglobin A_1c_ (HbA_1c_) reduction from a baseline of 10.2% to 8.5% after 12 months for an enrolled population of 137 patients. Emergency department visits decreased from year 1 to year 2 by 55 (22%), and hospitalizations by 62 (30%).

The concept of a peer as a CHW fits in the Appalachian culture. Although rural Appalachia has significant health disparities, it is an area with strong kinship networks that have traditions of neighbor helping neighbor. In rural Appalachia, CHWs must come from the community; otherwise, people will not receive them at home and will not be open to discussing their health conditions. CHWs form a close bond with patients; they become more open to change because of their relationship with the CHWs (22).

When the Centers for Medicare and Medicaid Services grant ended in 2015, we faced a dilemma. We had a model that demonstrated improved outcomes, but no payment model existed to sustain it. We realized that we needed to implement a 2-pronged strategy of seeking grant funding and concomitantly engaging health insurance payers to validate the model and establish an equitable payment model. Over a period of 2 years, we worked on grant funding and building relationships with Medicaid-managed care organizations (MCOs). Two milestones occurred that led to initiating this project. One was that the MCOs agreed to quarterly meetings for the purpose of monitoring the project. The second was that we obtained grant funding from the Appalachian Regional Commission and the Merck Foundation’s Bridging the Gap Initiative. The funding enabled us to replicate the model at a scale that would enroll a population large enough produce generalizable results. Because of our prior relationships with health centers in southern Ohio, eastern Kentucky, and West Virginia, we were able to enlist 11 federally qualified health centers (FQHCs) and 3 rural hospitals to replicate the model beginning in May 2017.

## Intervention Approach

The intervention strategy is to enroll high-risk patients with diabetes, heart disease, and chronic obstructive pulmonary disease (COPD) in a chronic care management model that includes CHWs on the team. The chronic care management (CCM) team at its core includes a mid-level provider, a nurse, and CHWs. Patient enrollment can happen in a few ways; the most common is through provider referrals. As providers identify patients that are struggling to control their chronic condition(s), they refer them to the CCM team. Referrals can also come from insurance partners who identify their high-risk members for referral or even occasionally from community partners.

**Figure 1:**
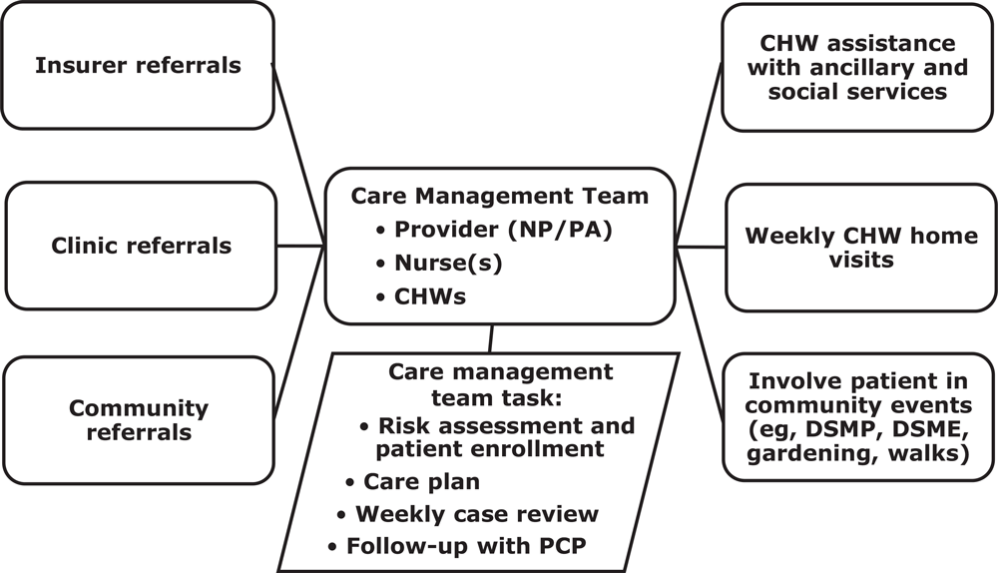
Organizational structure of the chronic care management team, implementation of community health worker–based chronic care management, rural Appalachia, United States, 2017–2019. Abbreviations: CHWs, community health workers; DMSE, diabetes self-management education; DMSP, diabetes self-management program; NP, nurse practitioner; PA, physician assistant; PCP, primary care provider.

The CCM team assesses patients’ level of risk and enrolls eligible patients in intensive care coordination (Figure 1). Once enrolled, the team works with the primary care provider to create care plans and regularly follows up with patients. 

The participants are patients of the health centers. Originally, we started with high-risk diabetes patients but then expanded to include patients with congestive heart failure and COPD because of the prevalence of these conditions and the fact that a high proportion of patients have these as comorbidities. Patients are eligible to enroll if they consent, including receiving home visits from a CHW.

The mid-level provider leads the weekly CCM team meetings, sometimes called “huddles.” They lead the process of reviewing and updating patients’ care plans. The nurse on the team manages the clinical side of case management, including tasks such as making referrals, contacting patients’ primary care providers, reconciling medicines, and helping to set up clinical appointments. The CHWs receive their instructions for patient care at the CCM team meetings and are in regular contact with the team nurse. They are full-time, permanent employees of the sponsoring clinics. Because patients have complex medical needs, the team’s nurse or mid-level provider directly supervise the CHWs. The CHW becomes the eyes and ears of the CCM team, bringing issues they see in the home back to the team, strategizing with the team to overcome barriers to compliance, and implementing those strategies with the patient.

The CHWs arrange a time to meet with patients in their homes on a weekly basis. In the home visit, the CHWs review the care plan with the patients, check on medication adherence, review and update self-management goals, and discuss issues that affect their lives. The most common issues that patients bring up are social, literacy, and economic barriers to their health. In one case, the person’s refrigerator stopped working, and she could not afford to replace it. As a result, her insulin spoiled. The CHW arranged for funding from a social service agency and her church to furnish her with a new refrigerator. In another case, the person did not know that he was eligible for low-income housing. The CHW referred him to the housing authority but on the next home visit realized that he needed help filling out the housing application, something that he was reluctant to admit.

As patients gain control over their condition, the CHWs reduce the frequency of the home visits. The CHWs, however, never drop patients; they keep them in their caseload. We learned this early on when the patients objected when CHWs told them that their condition was under control and they no longer needed to be in the program. Some threated to stop taking their medications so that the CHW would have to come back. Others simply refused to accept the CHWs decision and insisted they keep visiting them. We learned from experience that the social support of the CHWs is a strong motivating factor for patients to maintain control of their condition. We also learned that health insurance payers valued data that demonstrated patients’ continued control of their condition because of its implications for cost savings.

Consequently, we revised the caseload recommendations for CHWs. Initially CHWs’ caseload was 25 to 30 patients. As patients gained control, the CHWs reduced the frequency of home visits to once or twice per month (without dropping patients from their caseloads), which enabled them to take on new patients. With this combination of patients, CHWs have a caseload of 40 to 50 patients.

The organizational structure for CHWs in our model is based on local ownership. As employees of the health center, they adhere to norms and policies commensurate with their role. Nearly all CHWs are full-time employees. The exceptions are either because the CHW decided to seek further education while working part time or because the CHW was hired to work part time in the clinic as well as the community. The only 2 hiring criteria that we require are that CHWs be members of the community in which they work and that they have the ability to interact with patients with respect and empathy. The health centers add requirements such as medical assistant, nursing assistant, and prior experience in community service, based on their own hiring policies.

CHW training is multifaceted. CHWs complete the online training that is offered in each state, complemented by chronic disease self-management leader training, where they learn motivational interviewing, problem solving, and self-management skills. Each health center adds its own in-house training in technical skills, such as blood pressure monitoring, blood glucose monitoring, and spirometry. The CHWs most intensive training comes on the job from shadowing a veteran CHW, participating in the CCM team huddles and accompanying a nurse on home visits. Continuing education occurs every week in the CCM huddles. This is where they learn about managing diabetes, heart disease, and COPD. CHWs become highly knowledgeable in disease management through the practical experience of seeing what it takes for people to get their condition under control.

## Evaluation Method

For this study, we used data on enrollment in care coordination, and outcome data on HbA_1c_ of the patients who had diabetes. The CCM team documents patient enrollment when they consent to participate in this program. HbA_1c_ data are taken from patients’ medical records. On a quarterly basis, the health centers send de-identified data to Marshall University. We generate reports that show enrollment trends and changes in process and outcome measures. Each health center received a report, and we discussed quality improvement implications with them during quarterly site visits. For this study, we analyzed the change in HbA_1c_ measures from baseline to the most recent measure. 

We also used data to project cost savings of the patients with a baseline HbA_1c_ of 10% or more. In a recent study, the average hospitalization costs per patients with an HbA_1c_ greater than 10% was $4,000 per hospital visit (23). We used this finding to calculate potential annual savings of patients in our program. Our findings are from data on enrollment of high-risk patients, which has occurred continuously since May 2017.

## Findings

**Figure 2:**
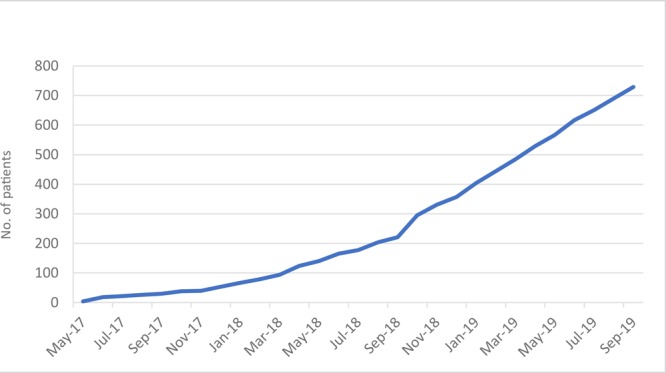
Enrollment in a community health worker–based chronic care management program, rural Appalachia, United States, 2017–2019.

As of September 2019, 729 high-risk patients have been enrolled. Enrollment began slowly, but as health care providers and patients became familiar with this model of care management, enrollment accelerated (Figure 2).

**Table:** 

Month and Year	No. of patients
May 2017	4
June 2017	18
July 2017	22
August 2017	26
September 2017	30
October 2017	38
November 2017	40
December 2017	53
January 2018	67
February 2018	79
March 2018	94
April 2018	124
May 2018	140
June 2018	165
July 2018	177
August 2018	204
September 2018	221
October 2018	295
November 2018	331
December 2018	357
January 2019	404
February 2019	444
March 2019	485
April 2019	529
May 2019	567
June 2019	617
July 2019	651
August 2019	690
September 2019	729

The most prevalent chronic condition of the enrolled patients was diabetes. Of these patients, 446 were enrolled long enough to have a baseline and at least a 6-month follow-up HbA_1c_ test. Patients whose HbA_1c_ improved during the study period had a mean decrease of 2.4 percentage points (Table). Of the total cohort, 63% (282 of 446) lowered their HbA_1c_ during the study period.

**Table:** Mean Change in HbA_1c_ of Participants (N = 446) of a Community Health Worker–Based Chronic Care Management Program, Rural Appalachia, United States, 2017–2017

Characteristic	No. of Patients	% of Cohort	Mean Baseline HbA_1c_, %	Mean Follow-Up HbA_1c_, %	Percentage-Point Difference
Decreased HbA_1c_	282	63	10.3	7.9	−2.4
Increased HbA_1c_	98	22	7.9	8.7	0.8
No change	66	15	8.7	8.7	0
Baseline HbA_1c_ ≥10% that lowered to <10%	96	22	11.9	8.2	−3.7

Abbreviation: HbA_1c_, hemoglobin A_1c_.

Data in the Table were used to project cost savings of the patients with a baseline HbA_1c_ of 10% or higher. Of the 96 patients who lowered their HbA_1c_ below 10%, we calculated a potential annual savings of $384,000, assuming a decrease of just one hospitalization per year for this cohort.

## Sustainability Strategy

Parallel to enlisting the health centers in the project, we built relationships with the Medicaid MCOs. Our approach was to invite them to learn about the model by observing how it was replicated in rural counties. After some months of relationship building, we initiated quarterly meetings with multiple types of health insurance companies (referred to as “payers”). In the meetings, we presented updates on enrollment and patients’ health status, enabling them to observe the project’s development. We have met quarterly since January of 2017. We receive technical assistance on these issues from the Center for Health Law and Policy Innovation at Harvard Law School. They attend the payers’ meetings and facilitate discussions on payment models and health care policy.

We progressed to the point that The Health Plan (THP) and Aetna Better Health of West Virginia signed a memorandum of understanding with of two of the FQHC partners, Community Care of West Virginia and Williamson Health and Wellness Center, to establish an equitable payment model for our CHW-Based CCM model. These 2 FQHCs are enrolling high-use THP and Aetna members in the CHW-Based CCM program. The payers are monitoring claims and health outcomes to document the cost savings.

We are using a graduated scale of collaborative funding where grant funding supports the startup costs of the CHWs’ salary and travel. Costs vary according to the salary scale of the health center and travel distances in each county. The approximate cost is $45,000 per CHW. As savings are realized, the payers have agreed to share the savings with FQHCs. In an early analysis, THP identified a savings of $5,000 per patient over a 4-month period. We are in the process of establishing a similar memorandum of understanding with 2 other health insurance payers in Ohio, which are in partnership with health centers that are implementing the CHW model.

## Discussion

A pattern in the health care agencies’ adoption of this model is that all but one are in rural areas; the exception is located in a low-income urban neighborhood. Adopting our model was a matter of identifying high-risk patients, organizing care coordination teams, and including CHWs on the teams. The concept of CHWs is accepted in Appalachia, and care coordination is widely practiced in the context of patient-centered medical homes. We brought these 2 concepts together with the aim of establishing a payment model that would make this relationship permanent. Evidence of acceptance of our model is in the adoption of the model by health centers in 3 neighboring states and the increased rate of enrollment over 29 months (Figure 2).

A factor that influenced the increase in the rate of enrollment was the emergence of physician champions in the health centers. Physician champions emerged as they observed how patients who had difficulties in managing their conditions would rapidly improve. This led physicians to be more assertive in identifying high-risk patients in their care and referring them to care coordination by the CHWs. Gradually, other providers in the practice would take notice and increase their referrals to the program. As one physician stated, “I hired a CHW, and now we work miracles” (24).

The impetus for the health centers to continuing enrolling patients is in part due to the improvement they see in the health status of their patients. A mean decrease in HbA_1c_ of 2.4 percentage points (Table) in a short period of time and that 60% or more of patients with diabetes lowered their blood glucose has been a stimulus to enroll as many high-risk patients as possible. Furthermore, when considering the outcomes for patients with a baseline HbA_1c_ of 10% and higher, the incentive is even greater. These outcomes contribute to achieving quality improvement incentives, which in turn increases their revenue.

A range of funders support this partnership, including national foundations, small private and family foundations, and hospital conversion foundations; the partnership is also supported by government grants. The grant funding covered the startup costs, thus enabling the health centers to begin as soon as they were ready. An indication of their commitment to the CHW model is that now they are adding CCM teams and CHWs beyond the scope of the grant funding.

A key component of this public–private partnership was the exploration of a Pay for Performance (PFP) model for sustainability of the project. PFP in health care is often referred to as value-based purchasing. PFP combines the concepts of a measured intervention to improve a societal condition (such as patient health outcomes), reduce costs, and improve care. These models are attached to a payment structure based on agreed-upon outcomes being delivered. Two funders, the Appalachian Regional Commission and the Benedum Foundation, consistently invested in the CHW model documented in this article to test such a PFP model. Although this program still uses the current fee-for-service structures in place in this country, it is building the case that such PFP models can be developed with the iterative process documented so that these innovative practices can be replicated with similar success in other practice settings. The results of this CHW model, along with the partnership between philanthropy and government funders, payers, and medical clinics, has documented the program model and has built a strong foundation for replication while reducing costs and significantly improving patient health outcomes.

### Implications for Public Health

Engaging the payers from the beginning of the project was a critical step. Their familiarity with the model grew as they observed its dissemination in a 3-state region. It was a natural progression from observation to their engagement in piloting payment models.

Initially, we expected to make a business case for the model by the end of the project. Because of the relationship with the payers, however, they saw the value of the CHWs and agreed to use their own data to make the business case. A milestone in this process was when THP agreed to enter into a memorandum of understanding with a health center to share data and collaborate in establishing an equitable payment model. This served as a template for other MCOs and health centers to pilot payment models. Our aim is that out of this collective experience there will be shared ownership of a payment model that will sustain CHW employment.

The initial CHWs focused primarily on patients with diabetes care because of our team’s previous work; however, as payers got more involved we began to focus CHW patient enrollment on patients with high health care costs. In addition, patient recruitment changed as payers became more involved. In the beginning, health centers identified patients, but we found that having payers identify high-cost patients offered the highest yield in improved outcomes and reduced costs.

Incorporating lay community members into chronic care management teams was a critical factor that established a direct link between the clinic and the community, resulting in care plans that addressed the medical needs and the social, economic, and self-management needs of the patient. Incorporating CHWs into the care management teams established a tangible connection between the clinic and the community. The resulting high proportion of patients that had better outcomes, along with reduction in emergency department and hospital visit rates, was then the basis for engaging payers to collaborate with health centers to pilot CHW-Based CCM payment models.
